# Toll-like 4 receptor /NFκB inflammatory/miR-146a pathway contributes to the ART-correlated preterm birth outcome

**DOI:** 10.18632/oncotarget.11987

**Published:** 2016-09-12

**Authors:** Xinqi Zhong, Yi-Zhou Jiang, Peiwen Liu, Wenzhi He, Zhongtang Xiong, Weijie Chang, Jiandong Zhu, Qiliang Cui

**Affiliations:** ^1^ Department of Pediatrics, The Third Affiliated Hospital of Guangzhou Medical University, Guangdong, China; ^2^ Institute for Advanced Study, Shenzhen University, Shenzhen, Guangdong, China; ^3^ Department of Pathogen Biology, School of Public Health and Tropical Medicine, Southern Medical University, Guangdong, China; ^4^ Experimental Department of Institute of Gynecology and Obstetrics, The Third Affiliated Hospital of Guangzhou Medical University, Guangdong, China; ^5^ Department of Pathology, The Third Affiliated Hospital of Guangzhou Medical University, Guangdong, China

**Keywords:** NFκB, inflammatory, miR-146a, assisted reproductive technology, preterm birth, Pathology Section

## Abstract

Assisted reproductive technology (ART) is widely used for the women with infertility conditions to achieve pregnancy. However, the adverse effects of ART may lead to poor perinatal and neonatal outcomes, e.g., preterm birth and low body weight. In this study, we investigated the inflammatory molecular factors and microRNA that might be involved in ART related preterm birth. We found the elevation of Toll-like 4 receptor (TLR4), activation of NFκB pathway and down-regulation of microRNA-146a (miR-146a), a negative regulator of NFκB, in the placenta of preterm birth and ART, indicating preterm birth and ART were associated with inflammation signaling activation. In vitro experiments demonstrated that miR-146a suppressed NFκB pathway and shifted the balance of cytokines in the cord blood toward a repertoire of pro-inflammatory outcomes by down-regulating IRAK1 and TRAF6. The pro-inflammatory cytokines IL-6, IFNγ and TNFα in the cord blood were highly expressed in the preterm and ART, while anti-inflammatory cytokine IL-10 was the lower in the preterm and ART. In summary, we firstly uncovered that TLR4/NFκB mediated inflammation signaling and miR-146a participated in ART-related preterm birth patients, which suggests that importance of TLR4/NFκB/miR-146a signaling in clinical interventions and biomarkers of ART-related perinatal or neonatal outcomes.

## INTRODUCTION

Assisted reproductive technology (ART) is an indispensable method for the infertile couples who want to have a baby. To date at least 5 million people were born under the help of ART^1^. However, emerging perinatal or neonatal problems such as low birth weight (LBW), preterm birth (PTB), small gestational age and birth defects are associated with ART [1]. ART is considered as an independent factor that contributes to the poor maternal and neonatal outcomes in pregnant women with reproductive disorders. The most frequently negative maternal outcomes are preterm birth, antepartum hemorrhage, and hypertensive disorders etc [2, 3]. For singletons, previous studies showed a higher risk in PTB and LBW for those who conceived with ART [4, 5]. Abnormal placentation was considered to be a crucial factor for poor obstetric outcomes in addition to other maternal malfunctions in endometrium [6, 7], myometrium [8] or cervix [9].

Altered inflammation mechanisms were found in placentas of women with reproductive disorders [10, 11]. For instances, placentas of obese women expressed high levels of cytokine profiles, e.g., IL-1, IL-6 and TNF-α [12]. Such a pro-inflammatory maternal and fetal environment plays a vital role in mediating adverse outcomes of pregnancy for both mother and fetus [13]. Previous report had found that placental inflammation was significantly raised in the mouse model of ART [14]. Furthermore, during manipulation of intra-cytoplasmic sperm injection, IL-6 mediated inflammation pathway was activated, possibly leading to the higher incidence of PTB and LBW [14].

Preterm labor occurring in more than 10% of all deliveries could be induced by inflammation [15]. Indeed, Elovitz etc have shown that administration of LPS (an agonist of Toll-like receptor 4, TLR4) could induce PTB in mice [16]. Other investigations also manifested that TLR4 agonist treatment enhanced NFκB phosphorylation [17] and subsequent secretion of pro-inflammatory cytokines (TNF-α and IFN-γ) [18]. However, the mechanism of poor outcomes of gestation (such as preterm birth) related with ART still need to be elucidated.

miRNAs are small non-coding RNAs that plays important functions in posttranscriptional gene transcription. Mis-regulations of miRNA expression often result in abnormal tissue homeostasis and cancers [18, 19]. miRNAs have been reported to be crucial factors in regulation of inflammation [20-23]. miR-146 and miR-155 were the most relevant microRNAs involving in the inflammation [21,23]. Studies have indicated that miR-146a regulates inflammation through TLR4-mediated pathway [20-23]. For example, expression of miR-146a was positively associated with those of IRAK, TRAF and TLR4 [24].

In this study, we test the hypothesis that TLR4-mediated signaling and miR-146a are important modulators of ART-associated PTB in the placenta.

## RESULTS

Patient information: To exclude the infection and disease induced inflammation, none of the study subjects with signs of infection and hypertension were recruited. Smokers were excluded. Patient ages were similar among ART/non-ART and full-term/preterm pregnancies. Gestational age of full-term was significantly higher than those pre-term pregnancies. Fetal weight of full-term was higher than preterm pregnancies. Maternal age, gestational age and birth weight were no significant different between non-ART and ART subjects.

### Immunohistochemistry study revealed an activating NFκB pathway in the preterm birth

**Figure 1:**
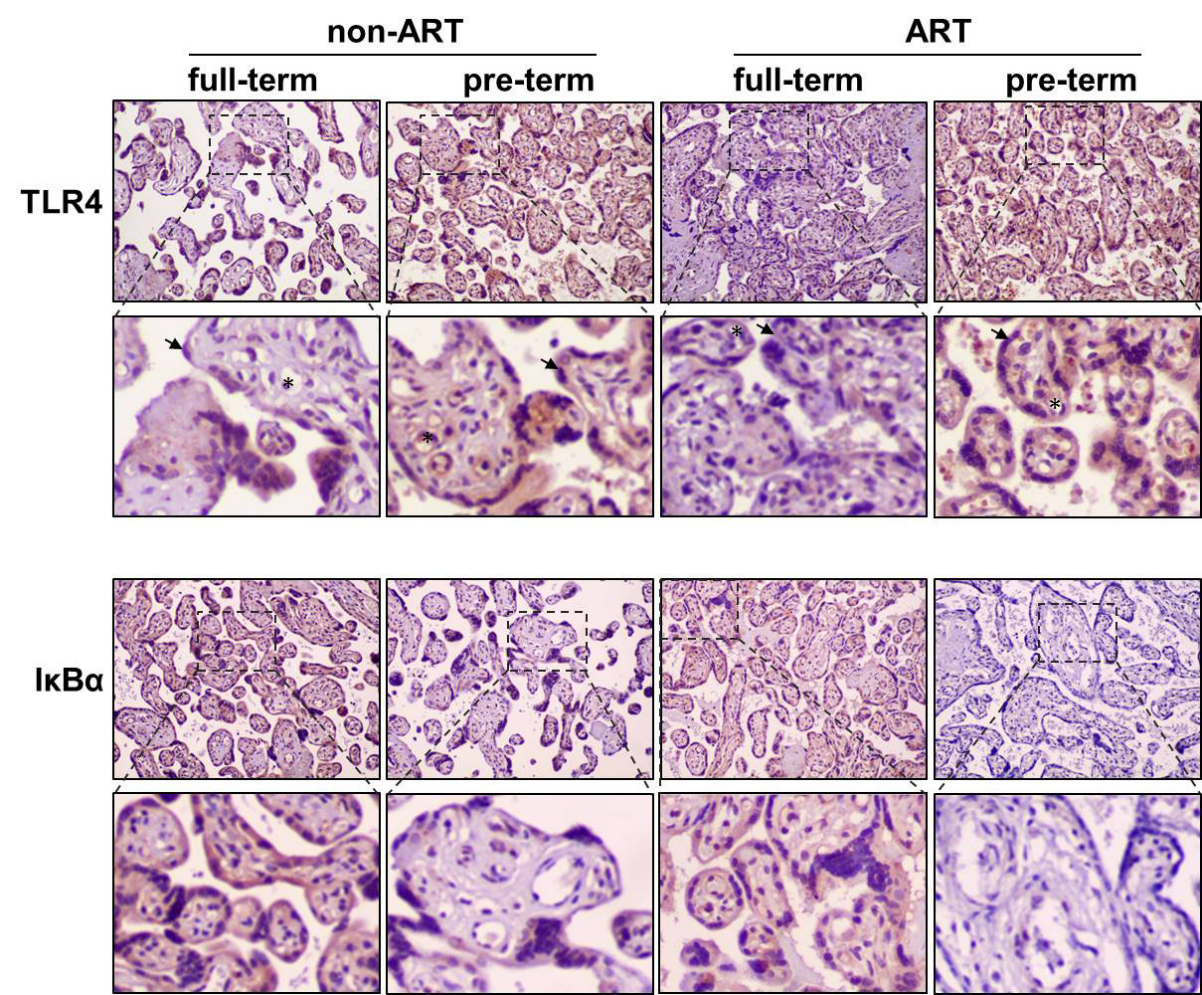
Immunohistochemical staining of TLR4 and IκBα in the placentas of ART/non-ART and full-term/preterm patients Horseradish pero xidase-conjugated anti-TLR4 or anti-IκBα antibodies were used to detect endogenous placental proteins. Representative images from three independent experiments (n =3 for each group) were shown. Brown color indicates expression of the targeted proteins. * indicates endothelial cells and arrow head indicates trophoblast cells.

TLR4 is a trans-membrane receptor that regulates innate immune response by activating NFκB pathway. NFκB signal activation depends on degradation of IκBα, an inhibitor of NFκB complex, whose degradation triggers the release of p65 for trans-locating into nucleus to regulate gene transcription. We explored whether the TLR4 and NFκB activation could be detected *in situ* by using immunohistochemistry. We found that TLR4 and IκBα were strongly expressed in trophoblast and endothelia of placental villi (Figure 1). Semi-quantification of the immunohistochemistry images was shown in Supplemental Figure 1. In both ART and non-ART subjects, TLR4 immunostaining intensity was higher in PTB as compared to the full-term. Consistently, NFκB negative regulator IκBα was lower in the preterm as compared to the full-term. Elevation of TLR4 and degradation of IκBα indicate the activation of NFκB pathway in preterm subjects. To further quantify the protein expression, placental tissue lysates were analyzed by Western-blot described as follows.

### The TLR4-mediated NFκB signaling activation contributed to ART and pre-term

**Figure 2:**
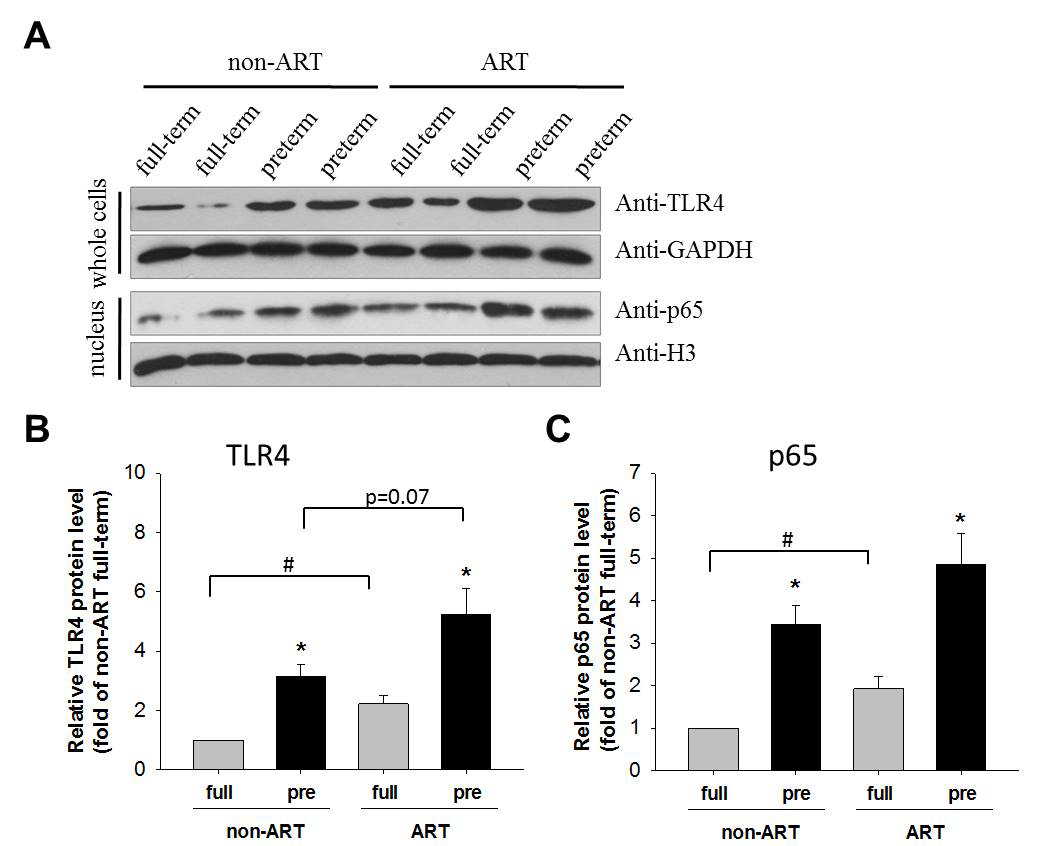
TLR4 and p65 protein in the placenta Representative Western-blot image of TLR4 and p65 in the placenta. Tissues separated from placenta were divided into two groups, one of which were lysed and detected by Western-blot using anti-TLR4 antibody, and the other of which were undergone nucleus-cytoplasm separation and the nuclear lysates were subsequently detected by anti-p65 antibody. GAPDH and H3 were used as a loading control for the cytoplasmic and nuclear proteins, respectively. Quantitative analysis of whole-cell TLR4. Data normalized to GAPDH were showed as Mean ± SEM and analyzed by Student's T-test (n=4). * *p*<0.05, pre-term vs. full-term; # *p*<0.05, non-ART vs. ART. Quantitative analysis of nuclear p65. Data normalized to H3 were showed as Mean ± SEM and analyzed by Student's T-test (n=4). * *p*<0.05, pre-term vs. full-term; # *p*<0.05, non-ART vs. ART.

The protein level of TLR4 and nuclear p65 were analyzed by Western blot in the placental tissues of ART/non-ART and full-term/preterm patients. TLR4 and nuclear p65 were higher in the preterm as compared to the full-term (Figure 2), confirming an enhanced inflammatory pathway in the preterm pregnancy. The consistent elevation of NFκB pathway (Figure 1 and 2) in PTB pregnancy was associated with lower birth weight (Table 1). Importantly, in the full-term subjects, ART further increased TLR4 expression and nuclear accumulation of p65 as compared to the non-ART control. In pre-term subjects, ART slightly (p=0.07) increase TLR4 protein levels. Because none of the study subjects had signs of infection, these data indicate that ART may be a contributory factor of inflammation.

**Table 1:** Patients demography information of ART/non-ART and full-term/preterm pregnancies and fetals (Mean ± SD)

	non-ART		ART	
	full	pre	full	pre
Mother's age (years)	32.5±2.1	33.8±2.2	36.8±3.8	35.0±2.9
Gestational age (weeks)	38.8±1.0	32.6±2.6*	38.4±2.2	35.4±0.9*
Birth weight (kg)	3.1±0.4	2.0±0.5*	3.2±0.4	2.4±0.3*

Student's T-test

* *p*<0.05, pre-term (pre) vs. full-term (full). N =4

### Association of miRNA and pre-term/ART birth

**Figure 3:**
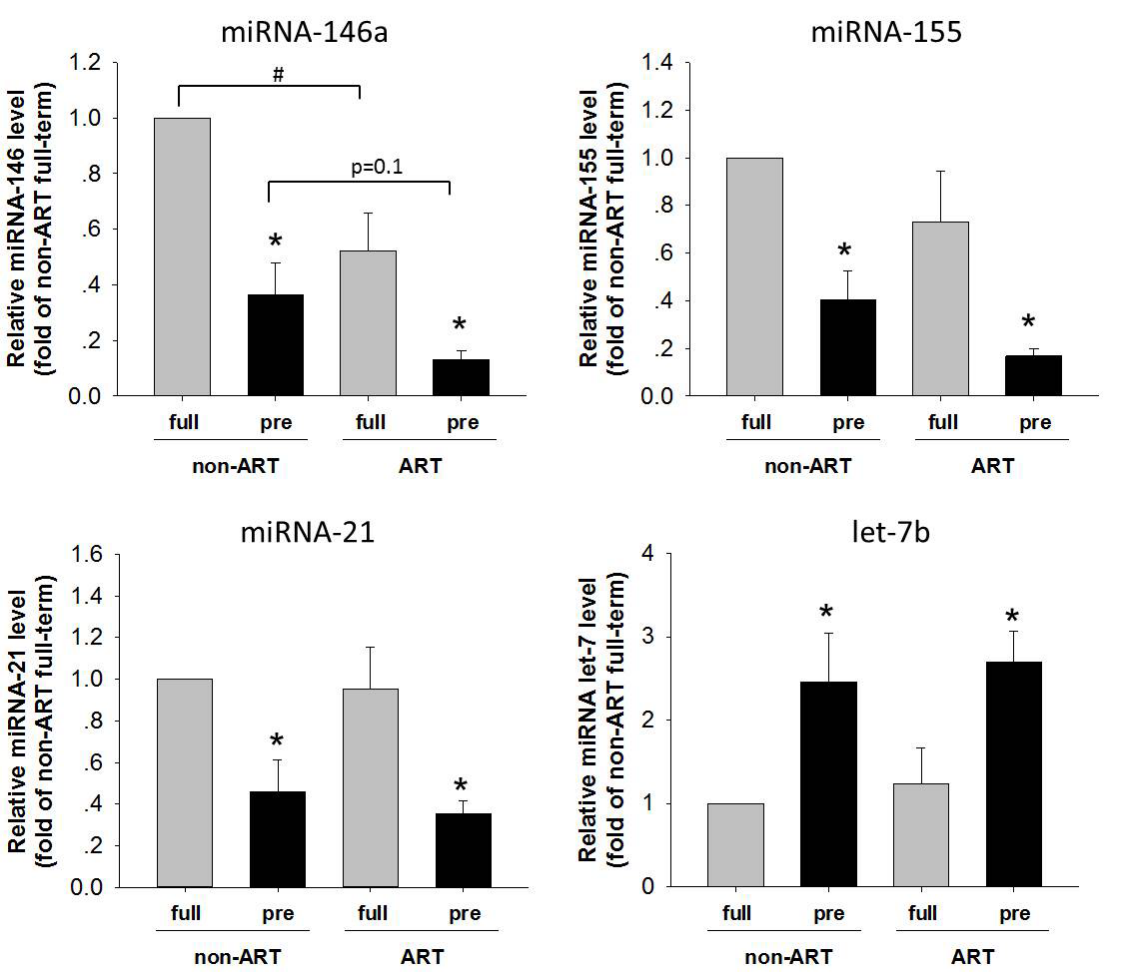
The levels of miR-146, miR-155, miR-21 and let-7b in the placentas of ART/non-ART and full-term/preterm qPCR was performed to quantify expression of miR-146, miR-155, miR-21 and let-7b in the placenta. Data normalized to U6 small nuclear RNA were shown as Mean ± SEM. and analyzed by Student's T-test (n=4). * *p*<0.05, pre-term vs. full-term; # *p*<0.05, non-ART vs. ART.

miRNAs have been found to regulate the complexity of NFκB pathway in human physiology and pathology. The miRNAs (miR-146a, miR-155, miR-21 and let-7b) regulate the components of NFκB pathway by direct binding and degradation of target mRNA. For instances, miR-146 inhibited pro-inflammatory cytokine secretion through IL-1 receptor-associated kinase [23]. miR-155 family were involved in the regulation of TLR4/NFκB pathway [20-22]. To assess the association of miRNAs in pre-term/ART birth, expression of miR-146a, miR-155, miR-21 and let-7b placental tissue was examined (Figure 3). Let-7 was increased in the pre-term than in the full-term placenta. In contrast, miR-146a, miR-155 and miR-21 were all significantly decreased in the pre-term than in the full-term. Importantly in the full-term placenta, miR-146a was further decreased in the ART as compared with that in the non-ART. These findings were in consistence with the activation of TLR4/NFκB in the pre-term/ART placenta (Figure 1).

### miR-146a Suppresses NFκB pathway in HTR-8/SV neo trophoblast cells

**Figure 4:**
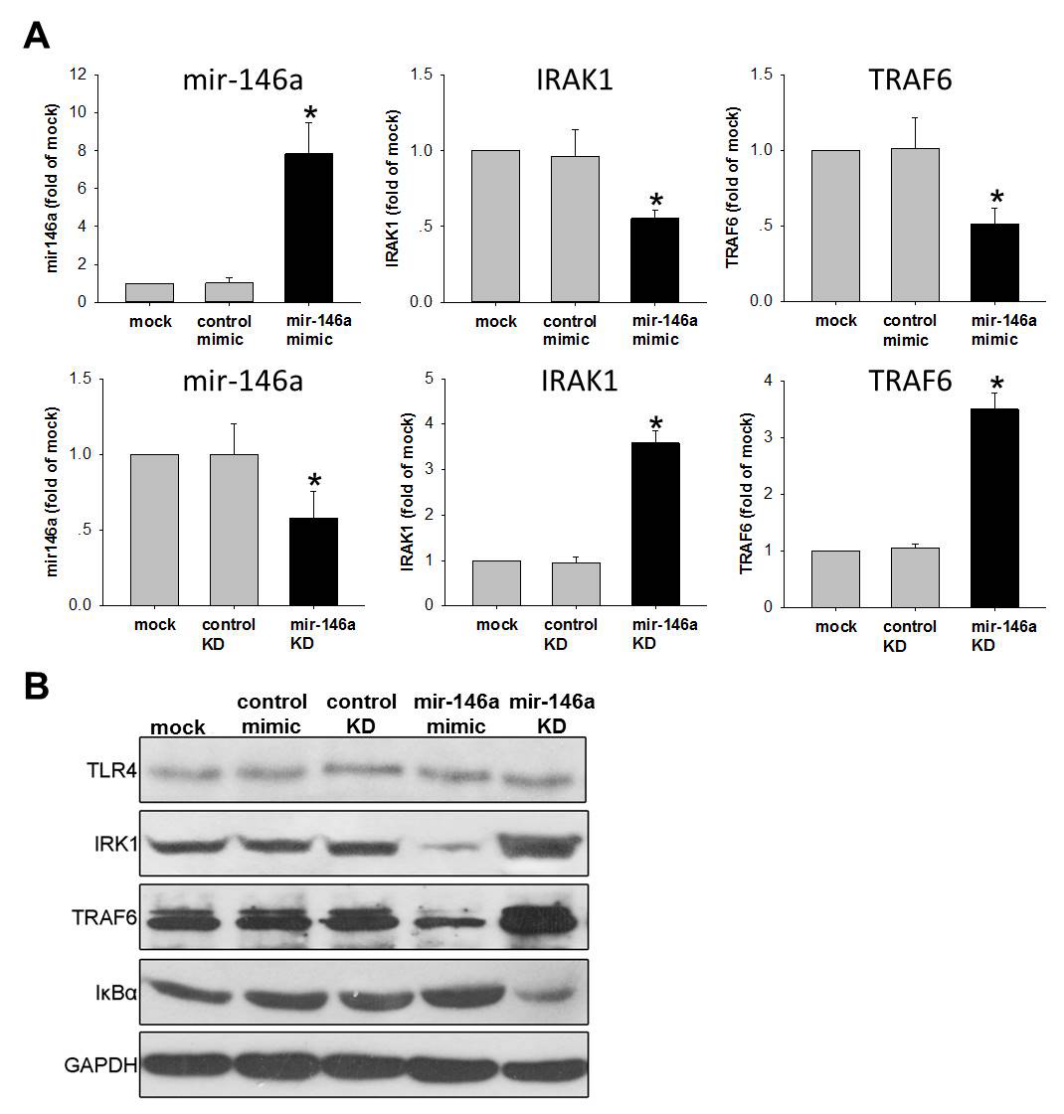
miR-146a inhibits NFκB pathway in human trophoblast cell line HTR-8/SV neo cells were transfested with 50nM mimic to increase miR-146a or transfested with 50nM anti-sense to knockdown (KD) miR-146a. Scrambled RNAs were used as control. (A) qPCR was performed to detect the level miR-146a and its down-stream targets IRAK1/TRAF6. Data were normalized to U6 small nuclear RNA or GAPDH mRNA. Normalized data were expressed as fold of mock control and shown as Mean ± SEM. (B) TLR4, IRK1, TRAF6 and IκBα protein were examined by Western-blot. GAPDH were used as loading control. Student's T-test (n=4), * *p*<0.05.

Decreased expression of miR-146a in the placenta was associated with pre-term/ART and NFκB (Figure 3). To test the causality, we investigated the gain-and-loss of function of miR-146a by mimic and antisense microRNA in human trophoblast cell that expressed TLR4/NFκB components. Increasing miR-146a levels led to the suppression of IRAK1 and TRAF6, while decreasing miR-146a caused the elevation of IRAK1 and TRAF6 (Figure 4). We further examined the expression of TLR4, IRK1, TRAF6 and IκBα protein in human trophoblast cells. Western blot clearly revealed that the protein levels of IRK1 and TRAF6 were significantly repressed when cells introduced with miR-146a mimic compared with those treated with control mimic. Conversely, the protein level of IκBα was dramatically induced in miR-146a treated cells. As a complementary, results from our knockdown assays confirmed that increased of IRK1 and TRAF6 protein levels in miR-146a depleted cells compared with cells transfected with control siRNA. While IκBα protein levels were downregulated.in miR-146a knockdown cells compared with that of control (Figure 4). Collectively, our data displayed that miR-146a can antagonize the activation of NFkB pathway in trophoblast cells.

### miR-146a plays crucial role of dysregulation between pro- and anti-inflammatory responses

**Figure 5:**
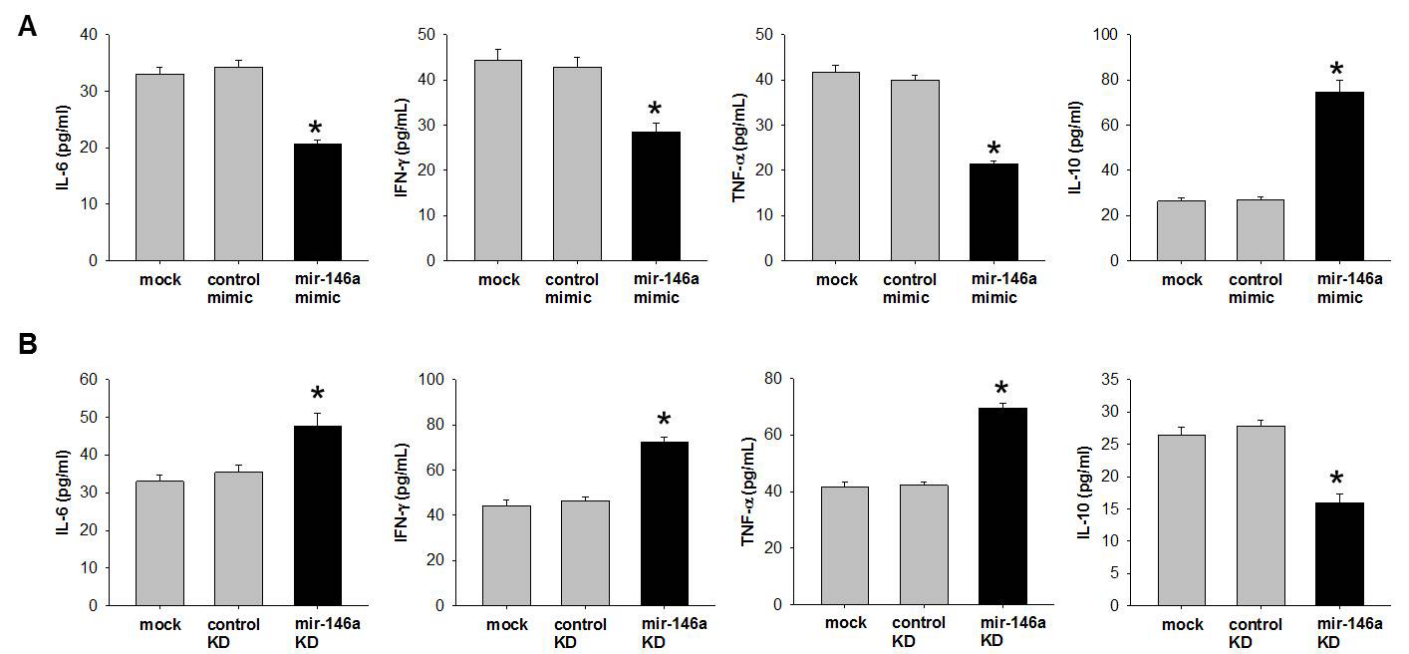
miR-146a inhibits pro-inflammatory cytokines and increase anti-inflammatory cytokine in human trophoblast cell line HTR-8/SV neo cells were (A) transfected with 50nM mimic to increase miR-146a or (B) transfected with 50nM anti-sense to knockdown (KD) miR-146a. Scrambled RNAs were used as control. After 48 hrs transfection, culture media were collected. Cytokines were quantified by ELISA. Data were shown as Mean ± SEM. Student's T-test (n=4), * *p*<0.05.

Activation of NFκB pathway and pro/anti-inflammatory cytokines reciprocally regulate each other [26, 27]. We then examined pro-and anti-inflammatory cytokines after manipulating miR-146a level in the trophoblast cell. Briefly, HTR-8/SV neo cells were either transfected with mimic or anti-sense of miR-146a and cultured for 48 hrs. Culture media were then collected for analysis of Cytokines profiling by ELISA. Our data showed that miR-146a mimic decreased expression of pro-inflammatory cytokines IL-6, IFNγ and TNF-α, while increased anti-inflammatory cytokine IL-10 compared with mock or control mimic (Figure 5). On the contrary, miR-146a knockdown increased secretion levels of IL-6, IFNγ and TNF-α, whereas decreased IL-10 compared with mock or control knockdown (Figure 5). Hence, miR-146a acts as an anti-inflammatory factor which maintains the balance between pro- and anti-inflammatory cytokines in the preterm and ART.

**Figure 6:**
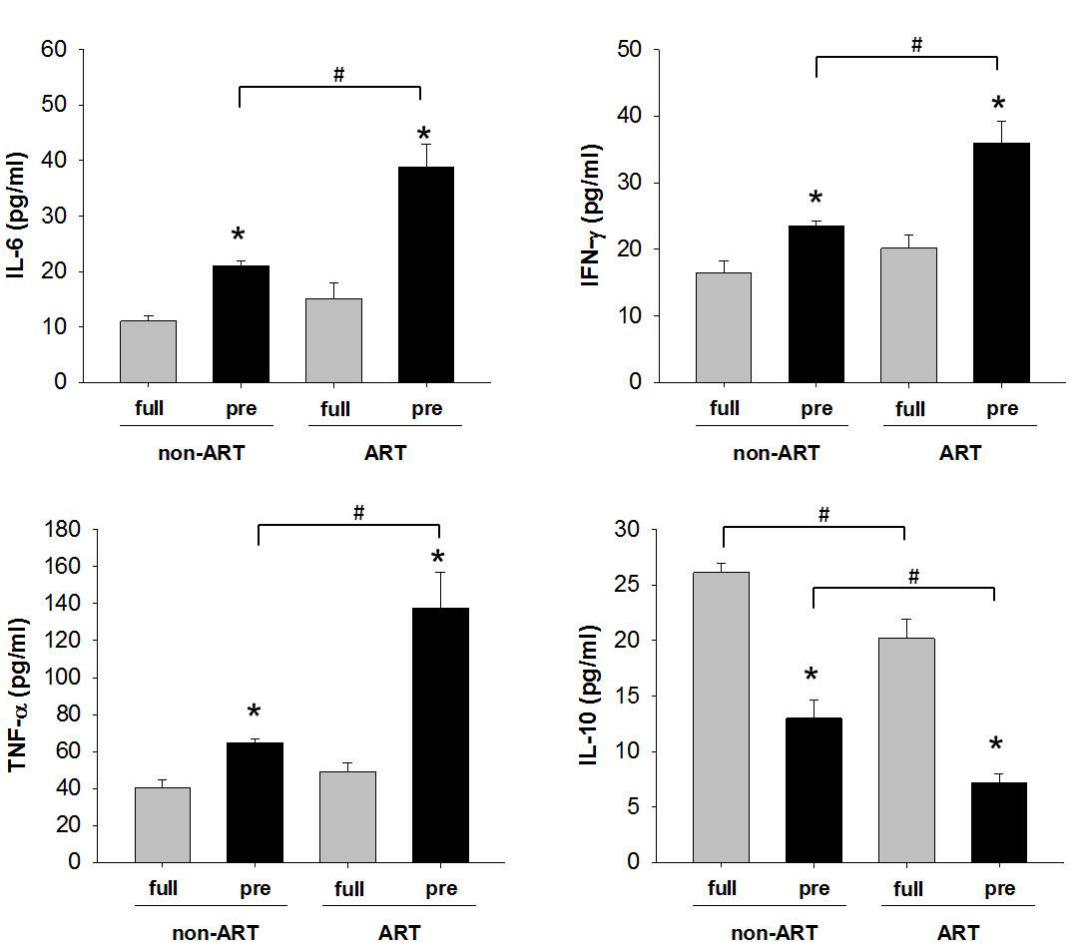
The expression of pro/anti-inflammatory cytokines in the umbilical cord blood of ART/non-ART and full-term/preterm ELISA was used to detect IL-6, IFN-γ, TNF-α and IL-10. The data were expressed as Mean ± SEM and analyzed by Student's T-test (n=4). * *p*<0.05, pre-term vs. full-term; # *p*<0.05, non-ART vs. ART.

To confirm *in vitro* finding, we examined the secretion of cytokines in the placental umbilical cord blood (Figure 6). The levels of pro-inflammatory cytokines IL-6, IFN-γ and TNF-α were higher in the preterm than those in the full-term subjects (Figure 3). The levels of anti-inflammatory cytokines IL-10 manifested a contrary pattern against pro-inflammatory cytokines. In full-term subjects, substantial down-regulation of IL-10 was found in ART as compared to non-ART subjects. These data indicate a shift of pro-inflammation in the pre-term fetus which may lead to pre-term birth. In addition, the protection of anti-inflammatory factor was withdrawn by unknown mechanism during or after ART process.

## DISCUSSION

Pregnancy and labor demonstrate the balance of the human immune system in which innate and adaptive immune response are controlled by pro-/ anti-inflammatory pathways that battle in the reproductive tissues (cervix and myometrium) and the maternal/fetal interface (placenta and decidual tissues). Premature activation of pro-inflammatory pathway can lead to a breakdown of fetomaternal immune-balance and play a critical role in the induction of labor, which subsequently can result in preterm birth (PTB) [28-30]. Preterm birth is the leading cause of neonatal morbidity and mortality. The biomarkers and therapeutic targets are still under investigation. Thus, we aimed to establish a relation between innate inflammation signaling and preterm birth. TLR4, a toll-like receptor responsible for activating the innate immune system, was tested. We also tested if ART was associated with preterm birth and inflammation.

As indicated in our results, by comparing with non-ART patients, NFκB signaling was robustly activated in response to TLR4 up-regulation in preterm birth patients with ART (Figures 1 and 2). Because NFκB was a transcriptional factor that took part in many cellular process like cell proliferation, cell cycle progression, apoptosis and inflammation [31-34]. Taken preterm birth-related inflammation for consideration, the potential inflammatory factors potentially regulated by NFκB were of our interests for monitoring. Numerous studies supported the association between elevated levels of circulating pro-inflammatory cytokines and PTB [35-36], specifically, IL-1, IL-6 and TNF-α might be the major players for the onset of PTB [37-40]. In contrary to pro-inflammatory cytokines, anti-inflammatory cytokines like IL-10 had been shown a decrease in gestational tissues [41], suggesting IL-10 in down-regulating inflammatory responses. In our study, we detected pro- and anti-inflammatory cytokines from cord blood of ART and non-ART patients. Among all patients with cesarean section, results indicated that the pro-inflammatory cytokines including IL-6, IFN-γ and TNF-α were higher in ART patients than in non-ART patients, meanwhile, anti-inflammatory cytokines IL-10 were all significantly lower in ART patients than non-ART patients (Figure 3). These data suggest that auto-immune or unknown factor-driven inflammation were a major cause for the ART-related preterm birth outcome.

miRNAs were reported to be a key factor in regulating inflammation process [20-23]. For example, miR-146a in cartilage has long been associated with osteoarthritis42. Moreover, under chronic inflammatory stimulation, miR-146a is overexpressed in different cell types, including endothelial cells and white blood cells [43]. High level of miR-146a was found in the blood of patients with coronary artery disease [24]. In the meantime, expression of miR-155 is elevated significantly in the serum and atherosclerotic lesions of atherosclerosis patients [44]. Importantly, miR-155 has also been shown as an oncogenic pro-inflammatory microRNA that is up-regulated in a number of malignancies [45, 46]. Studies have indicated that miR-146a regulates inflammation through TLR4-mediated pathway [20-23]. For example, expression of miR-146a was positively associated with those of IRAK, TRAF and TLR4 [24]. Although the exact mechanism by which miR-146a and miR-155 expression contributes to inflammation is not clear, study has shown miR-146a targets a wide variety of genes involved in inflammation, oxidation, and apoptosis [47]. Our results suggested that both miR-146a and miR-155 participated in repression of inflammatory responses in ART-related preterm birth.

Our findings above could explain ART-resultant inflammation itself was an independent reason for the ART-patients with poor obstetrical or neonatal outcomes. Further investigations on the regulation of inflammation signaling could be a potentially therapeutic intervention in ART patients.

## MATERIALS AND METHODS

### Patients and tissues

Patients were recruited from obstetrics and gynecology department, 3^rd^ affiliated hospital of Guangdong Medical University, China. Patients had no preeclampsia, no insulin treatment and no infection in the urinal duct, pelvis, periodontitis, respiratory tract and myocarditis during gestation. Recurrent miscarriage, premature rupture and usage of antibiotics during the pregnancy or prenatal period were all excluded. The patients received *in vitro* fertilization pre-embryo transfer only because of low sperm count or fallopian tube obstruction/removal. All the bio-samples were collected with the consent of patients and their direct relatives. The experiments protocols were approved by the ethic committee of the hospital. The placentas, amniotic fluid and cord blood were collected immediately after the C-section deliveries and stored in cryo-refrigerator for biochemical, immunohistochemistry and ELISA experiments. The full-term samples were collected from non-medically indicated C-section. The pre-term samples were collected from medically indicated C-section.

### Reagents and antibodies

TRIzol for RNA extraction was purchased from Invitrogen. GAPDH antibody (Cat#5174), IκBα antibody (Cat#4814, Cell signaling technology, USA), and p65 antibody (Cat#8242) were purchased from Cell Signaling Technology (USA). Histone 3 antibody (#SC10809) was purchased from Santa Cruz Company (USA). TLR4 (Cat# AB47093) was purchased from ABCAM Company.

### Immunohistochemistry

Paraffin-embeded embedded Placental tissues were cut for 5μm each slice in microtome. After deparaffinization in xylene, rehydration and blocking, the tissue sections were probed with anti-TLR4 and anti-IκBα antibodies (1:100) for 1 hour. Pre-immune IgG was used as negative control. Immunoreactions were detected using the diaminobenzidine reagent, followed by counterstaining with hematoxylin. At last, the slices were visualized under an inverted microscope (Olympus IX71, Japan).

### Western-blotting

Western-blot was performed as previously described [25]. Placenta tissues were collected 5 cm away from the umbilical cord insertion site. For the whole cell lysis, 1 cm3 placental villi from beneath the chorionic and basal plates were mechanically homogenized, then lysed in TSPI buffer [50 mM Tris-HCl (pH7.5), 150 mM NaCl, 1 mM EDTA, 10 mg/ml of leupeptin, 1 mg/ml of aprotinin, 10 mg/ml of pepstatin, 1% NP-40 and 0.5 mM Pefabloc SC]. To separate cytoplasmic and nuclear protein, homogenization was performed by applying 15 strokes with a 27 gauge needle on ice. 50μL of 2.5M sucrose was added to restore isotonic conditions. The first round of centrifugation was performed at 6300g for 5min at 4°C. The pellet washed with TSE buffer (10mM Tris, 300mM sucrose, 1mM EDTA, 0.1% NP40, PH7.5) at 4000g for 5min at 4°C until the supernatant was clear. The resulting supernatant was discarded and the pellets were nucleus.

The proteins were separated by 12% SDS PAGE and transferred onto PVDF membrane (Millipore). After incubation with primary antibodies (1:2000) overnight at 4oC, the secondary HRP-conjugated antibodies (1:2000) and ECL detection kit (Amersham Pharmacia Biotech) were used to visualize target proteins. The images of westernblot were semi-quantified by Image J imaging analysis software (National Institutes of Health; Bethesda, MD). The integrated OD value for each band was calculated as: OD _(antibody staining)_ – OD _(background)_.

### Enzyme-linked immune-sorbent assay (ELISA)

The concentration of cytokines (IL-6, IFN-γ, TNF-α and IL-10) in cord blood of the patients and cell culture media were determined using ELISA kit (Elisakit, Australia) according to the manufacturer's instructions.

### Quantitative real-time PCR (qPCR)

The total RNAs were extracted from placental tissues by a Trizol-chloroform method (Trizol, Invitrogen USA, Cat#19996-026) from the placentas tissues. Quantitative real-time PCR was performed in StepOne Plus (Life Technologies, USA) by using SYBR Premix Ex Tag kit (Takara, Japan) according to the manufacture's protocols. The qPCR primers and sequence were: miR-21-RT GTCGTATCCAGTGCAGGGTCCGAGGTATTC GCACTGGATACGACTCAACA; miR-21-F GCGCTAGCTTATCAGACTGA; miR-146a-RT GTCGTATCCAGTGCAGGGTCCGAGGTATT CGCACTGGATACGACAACCCA; miR-146a-F GCGTGAGAACTGAATTCCA; miR-155-RT GTCGTATCCAGTGCAGGGTCCGAGGTA TTCGCACTGGATACGACACCCCT; miR-155-F GCGTTAATGCTAATCGTGAT; let-7b-RT GTCGTATCCAGTGCAGGGTCCGAGG TATTCGCACTGGATACGACAACTAT; let-7b-F GCGCTGAGGTAGTAGGTTGT; U6-F CTCGCTTCGGCAGCACA; U6-R AACGCTTCACGAATTTGCGT; Universe-R GTGCAGGGTCCGAGGT.

### Cell culture and transfection

Human trophoblast cell line HTR-8/SV neo cells were cultured in RPMI 1640 media with 10% FBS and 1% P/S. Cells at 40-50% confluence were transfected with miR-146a (sence: 5′UGAGAACUGAAUUCCAUGGUU3′, anti-sence:5′CCCAUGGAAUUCAGUUCUCAUU3′) or mimic(sence:5′UUCUCCGAACGUGUCACGUTT3′,anti-sence:5′ACGUGACACGUUCGGAGAATT3′) by riboFECT (Guangzhou RiboBio, China) according to manufacturer's instruction. After 24 hrs of transfection, total RNA was isolated. qPCR was used to examine miR-146a levels and its target mRNAs eg. IRAK1 and TRAF6. After 48 hrs of transfection, cell lysates and culture media were collected to quantify protein levels.

### Software and statistical analysis

The data were analyzed by Student's T-test.

## SUPPLEMENTARY MATERIALS FIGURE


