# Supplementary material for: Toll-like 4 receptor /NFκB inflammatory/miR-146a pathway contributes to the ART-correlated preterm birth outcome
Source: Oncotarget. 2016 Sep 12;7(45):72475–85. doi: 10.18632/oncotarget.11987 (PMC5341923; doi:10.18632/oncotarget.11987)
Supplement: Supplementary file 1 [file oncotarget-07-72475-s001.pdf]

Toll-like 4 receptor /NFκB inflammatory/miR-146a pathway contributes to the ART-correlated preterm birth outcome

Supplementary Material

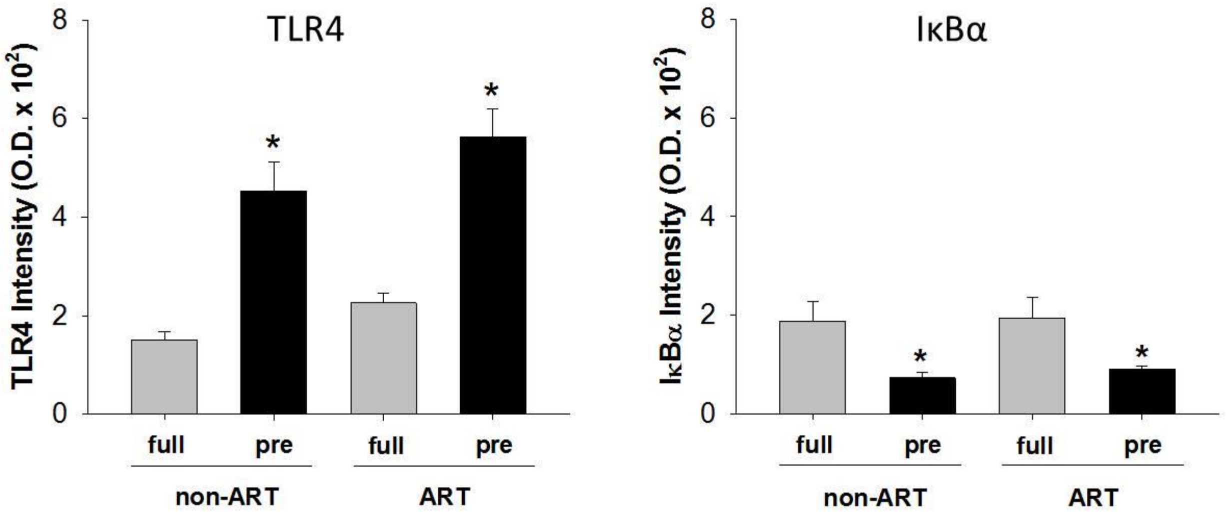

Supplemental Figure 1. To semi-quantitatively compare the antibody staining intensity in tissues, images were taken at exactly the same exposure time and light intensity. The images were uniformly converted to 8-bit gray-scale images. For each tissue, the optical density (OD) value was measured by the Image-J imaging analysis software (National Institutes of Health; Bethesda, MD). The OD value for each tissue core was calculated as: OD (antibody staining) - OD (preimmune rabbit IgG). The tissue sections were then counterstained with Harris Modified Hematoxylin (Fisher Scientific), and the images were recorded again for qualitative analysis.
